# Asymptomatic Malaria Cases and *Plasmodium* Species in Mainland Tanzania and Zanzibar Archipelago (Pemba)

**DOI:** 10.3390/pathogens13121140

**Published:** 2024-12-23

**Authors:** Daria Kołodziej, Wanesa Wilczyńska, Małgorzata Marchelek-Myśliwiec, Dariusz Świetlik, Heriel Zacharia Ammi, Mohamed Othman Athumani, Krzysztof Korzeniewski

**Affiliations:** 1 Department of Epidemiology and Tropical Medicine, Military Institute of Medicine—National Research Institute, 128 Szaserów St., 04-141 Warsaw, Poland; dkolodziej@wim.mil.pl (D.K.); wanesa.wilczynska@gmail.com (W.W.); 2 Clinic of Nephrology, Transplantology and Internal Medicine, Pomeranian Medical University, 70-111 Szczecin, Poland; malgorzata.marchelek@gmail.com; 3 Department of Biostatistics and Neural Networks, Medical University of Gdańsk, 1 Dębinki St., 80-211 Gdańsk, Poland; dswietlik@gumed.edu.pl; 4 Karatu Lutheran Hospital, Karatu District, Karatu Town P.O. Box 165, Arusha Region, Tanzania; herizammi@yahoo.com; 5 Amal Hospital, Chake Chake 74204, Pemba, Zanzibar Archipelago, Tanzania; othman.dr@outlook.com

**Keywords:** malaria, *Plasmodium*, Africa, mainland Tanzania, Zanzibar Archipelago

## Abstract

Malaria remains a major public health threat in Sub-Saharan Africa. According to the World Health Organization (WHO) estimates, *Plasmodium falciparum* species account for nearly 100% of the malaria cases occurring on the African continent. According to the Centers for Disease Control and Prevention (CDC), falciparum malaria predominates, but non-falciparum species are also present in Africa. The aim of the study was to assess the occurrence of asymptomatic malaria cases, as well as to identify *Plasmodium* species at two different settings with the lowest index of infections in Tanzania (according to the Tanzanian Ministry of Health < 1%), i.e., on the mainland (Arusha Region) and on the Pemba Island (Zanzibar Archipelago). The study was conducted in June 2023 and involved 722 individuals, including 449 residents of mainland Tanzania and 273 residents of the Zanzibar Archipelago. The screening consisted of two phases. In the first one, which was carried out at two different settings, i.e., in the Karatu Lutheran Hospital (Arusha Region, mainland Tanzania) and the Amal Hospital (Pemba, Zanzibar Archipelago), mRDTs (malaria rapid diagnostic tests) were performed, haemoglobin concentrations were measured, and blood samples for further molecular tests were collected onto Whatman micro cards from each of the individuals involved. In the second phase (conducted in Poland, Europe), RT-PCR tests for malaria were performed. The screening found asymptomatic *Plasmodium* infections in 4.2% of the study subjects from mainland Tanzania and in 4.8% from the Archipelago. The research confirmed cases of *P. falciparum* malaria but also found single cases of mixed infections with *P. falciparum* + *P. malariae* or *P. ovale*. The results demonstrated that the occurrence of malaria in northern mainland and Zanzibar Archipelago is higher than the official MoH reports present. The study findings are consistent with the reports by CDC, which suggest that non-falciparum species are also present in Sub-Saharan Africa.

## 1. Introduction

Malaria is a vector-borne disease transmitted by female *Anopheles* mosquitoes. There are five *Plasmodium* parasite species which cause malaria in humans: *P. falciparum* (responsible for the most severe form of the disease), *P. vivax*, *P. malariae*, *P. ovale* and *P. knowlesi* [1]. The signs and symptoms of symptomatic malaria include fever, chills, headache and general weakness or discomfort; the prognosis worsens as the parasite count rises. In some scenarios, the symptoms can be severe, leading to serious complications such as hypoglycaemia, acute liver failure, jaundice, pulmonary oedema, CNS disorders, and, eventually, death [2]. A substantial proportion of malaria cases in Africa are asymptomatic, which may be the result of the continuous exposure to *Plasmodium* species and a gradual acquisition of protective immunity. Such naturally acquired immunity against malaria not only significantly lowers the risk of a re-infection but also prevents severe complications [3]. On the other hand, a high rate of asymptomatic malaria cases in Africa contributes to a rise in clinical cases and poses a major obstacle to eliminating the disease in the region. In 2022, of the 249 million malaria cases reported globally, as much as 94% were reported to be from Africa. Tanzania is an example of a country where *Plasmodium* infections are at a record high, with 3.6 million malaria cases reported in 2023 alone. *Plasmodium falciparum* is the most prevalent human malaria parasite in Sub-Saharan Africa. According to the World Health Organization (WHO) estimates, *Plasmodium falciparum* species are responsible for nearly 100% of malaria cases on the African continent [2]. The position of the Centers for Disease Control and Prevention (CDC) also confirms the dominance of *P. falciparum* in Africa but emphasises the occurrence of other non-*falciparum* species, including *P. malariae*, *P. ovale*, and *P. vivax*, as well [4]. The Ministry of Health (MoH) in Tanzania has estimated that the Arusha Region (northern parts of mainland Tanzania) and the Zanzibar Archipelago have the lowest malaria prevalence of all the regions in the country [5]. The latest reports from the Tanzanian MoH suggest that the malaria prevalence rate in those two regions has dropped to <1% in recent years [6]. Despite the educational activities conducted by health services, knowledge about malaria transmission and effective preventive measures (bed net use) among Tanzanians seems to be moderate, and the access to health care is limited due to the low funding of the public health sector. Thus, the risk of spreading and the difficulty in controlling malaria may be exacerbated by the high percentage of asymptomatic cases in the local population.

The aim of the present study was to assess the actual occurrence of asymptomatic malaria cases, as well as to identify *Plasmodium* species responsible for malaria at the two selected settings, i.e., on the mainland (Arusha Region) and on Pemba Island (Zanzibar Archipelago), and compare this information with the official data provided by the MoH Tanzania, WHO and CDC.

## 2. Materials and Methods

### 2.1. Study Groups

**Figure 1:**
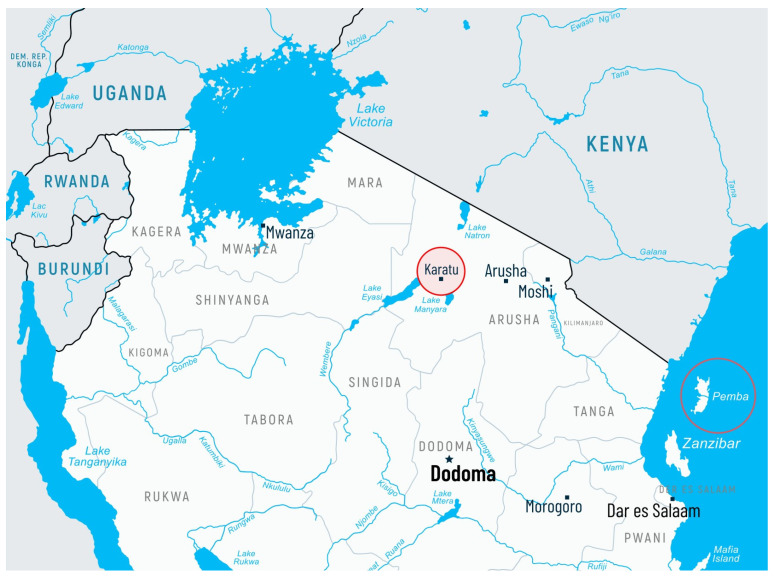
Map of northern Tanzania showing the location of Karatu Town (mainland Tanzania) and Pemba Island (Zanzibar Archipelago).

The present study was conducted in June 2023 and involved a total of 722 individuals of both sexes (females and males) at different ages (children and adults), who were residents in the pre-selected settings, i.e., Karatu District in the Arusha Region (northern parts of mainland Tanzania) and Pemba Island (Zanzibar archipelago) (Figure 1).

The first study group consisted of 449 randomly selected residents of the Karatu District (with an area of 3207 km^2^ and a population of 280,000) located in the Arusha Region, aged between 1 and 96 years old, who volunteered to take part in the study at the Karatu Lutheran Hospital in Karatu (a town with a population of 26,000 people lying at 1420 m above sea level, located 30 km from the Ngorongoro Conservation Area, which is the gateway to the Serengeti National Park). Participants were asked to fill in patient questionnaires and sign an informed consent form in Swahili (for paediatric patients, informed consent was obtained from the children’s parents or their legal guardians).

The second study group comprised 273 randomly selected residents of the Pemba Island, which is part of the Zanzibar Archipelago (with an area of 988 km^2^ and a population of 543,000), aged between 1 and 85 years old, who volunteered to take part in the study at the Amal Hospital in Chake Chake Town (a town on an island on the Indian Ocean with a population of 52,000 residents, lying at 0 m above sea level, 50 km off the Tanzanian mainland). Participants were asked to fill in patient questionnaires and sign an informed consent form in Swahili (for paediatric patients, informed consent was obtained from the children’s parents or legal guardians).

The inclusion criteria of the patients participating in the study were determined as follows: no clinical signs and symptoms of malaria, body temperature ≤ 37.5 °C, and non-use of antimalarial medications during the 4 weeks prior to the commencement of the study. Patients with difficult venous access were excluded from this research study. Patients from both groups had their blood samples taken and were tested for malaria using rapid diagnostic tests (positive mRDTs results were confirmed by light microscopy); they also had their haemoglobin (Hb) concentration measured using a portable Hb analyser. Next, the same blood samples which had been used for Hb measurements were applied onto Whatman micro cards for further molecular biology diagnostics. Patients diagnosed with the Plasmodium infection in the first phase of the study (mRDT + microscopic confirmation) received antimalarial treatment. The second phase of the study was conducted at the Department of Epidemiology and Tropical Medicine (DETM) in Poland and consisted of performing molecular tests (RT-PCR) to confirm or rule out malaria infections, and to identify the Plasmodium species responsible for the infections.

### 2.2. Malaria Screening

Immunochromatographic rapid tests (Pf/Pv/Pan; Beright, Hangzhou, China, Alltest Biotech Co. Ltd.) for the detection of four different *Plasmodium* species (*P. falciparum*, *P. ovale*/*P. malariae*, *P. vivax*) were used. mRDTs were performed on venous blood collected from subjects with no clinical symptoms of malaria. The rapid test sensitivity was 98.7%, and its specificity—99.3% (according to the manufacturer) [7].

Molecular diagnostics by RT-PCR. Dried blood specimens (200–300 µL of venous blood) were collected on the Whatman micro cards [8], and next, transported to the DETM in Poland. The genetic material was isolated using the Sherlock AX Kit (A&A Biotechnology, Gdańsk, Poland) in line with the manufacturer’s instructions (a test for the manual isolation of genomic DNA working on the principle of nucleic acid absorption on ion-exchange membranes, combined with DNA precipitation with isopropanol) [9]. The isolated DNA was suspended in 100 µL of the TE buffer (buffer solution used for elution and storage of nucleic acids after isolation; consists of Tris-HCl and EDTA). RT-PCR diagnostics was performed using the VIASURE Malaria RT-PCR Detection Kit for the qualitative detection of *Plasmodium falciparum*, *P. malariae*, *P. ovale* and *P. vivax* DNA [10]. RT-PCRs were run on an AriaMx RT-PCR system (Agilent Technologies, Santa Clara, CA, USA).

### 2.3. Haemoglobin Measurements

Haemoglobin concentration was measured using a portable DiaSpect^TM^ analyser (EKF Diagnostics, Cardiff, UK). Haemoglobin levels were interpreted in line with the WHO criteria: mild anaemia (11.0–11.9 g/dL in women, 11.0–12.9 g/dL in men), moderate anaemia (8.0–10.9 g/dL), and severe anaemia (<8.0 g/dL).

### 2.4. Statistical Methods

The StatSoft Inc. (2014) STATISTICA version 12.0 (StatSoft Polska Sp. z o.o., Kraków, Poland) [11] and an Excel spreadsheet were used for all calculations. The significance of the differences between the *Plasmodium*-infected vs. non-infected individuals (unpaired variables model) was tested using the significance of differences test (Student’s *t*-test or Mann–Whitney U test). The chi-square test for independence was used to test for qualitative variables (using Yates correction for cell frequencies below 10, Cochrane’s test or Fisher’s exact test, as appropriate). In all calculations, the level of statistical significance was set at *p* = 0.05. To determine the impact of the selected parameters on a positive test result for *Plasmodium* infection, univariate and multivariate regressions were used. In the multivariate analysis, only statistically significant parameters obtained in the univariate analysis were included.

### 2.5. Ethical Approval

The research project entitled ‘Prevalence of malaria in northern Tanzania in the context of the necessity of administration of anti-malarial chemoprophylaxis among European travellers’ was obtained from the Ministry of Health and the National Institute for Medical Research, Dar es Salaam, the United Republic of Tanzania (Ethical Clearance Certificate for Conducting Medical Research in Tanzania, Ref. No. NIMR/HQ/R.8a/Vol IX/4040, 5 July 2022). Parental consent was obtained for each child to participate in the study. The collection of blood samples was conducted and supervised by the medical personnel employed at the Karatu Lutheran Hospital (Karatu Town, mainland) and the Amal Hospital (Chake Chake Town, Pemba Island, Zanzibar Archipelago).

## 3. Results

The study involved a group of 722 asymptomatic patients of both sexes, including 107 children, aged 1–15 years, and 615 adults. The sample was divided into 2 groups, depending on the place of residence; the first group consisted of residents of the Karatu District, Arusha Region in mainland Tanzania (*n* = 449) and the other group involved the residents of the Pemba Island in the Zanzibar Archipelago (*n* = 273).

Malaria rapid diagnostic tests (mRDT) revealed a total of five *Plasmodium* infections (two *P. falciparum*, two *P. ovale*/*P. malariae*, one *P. falciparum* + *P. ovale*/*P. malariae*) in the first group (i.e., the group consisting of 449 patients tested at the Karatu Lutheran Hospital, mainland Tanzania), and positive tests accounted for 1.1% of all the tests performed in this group; all positive mRDT results were verified by microscopic examination performed by qualified personnel at the Karatu Lutheran Hospital. During phase II of the study, all samples were tested using molecular methods (RT-PCR). Molecular tests found a total of 19 *Plasmodium* infections (including those infections which had been detected using mRDTs), and positive tests accounted for 4.2% of all the tests performed in this group. In two cases, RT-PCR detected *P. falciparum*, although mRDT detected Pan species only. RT-PCR is more sensitive than mRDT and detects infections with low parasitemia, which may be undetectable by the less sensitive mRDT. *P. vivax* infection was not detected in any of the positive results. All of the study participants who tested positive for malaria were adults, and two of those infected were pregnant women. In the group of *Plasmodium*-infected individuals, only one female patient had a normal haemoglobin concentration, two patients had mild anaemia, six patients had moderate anaemia, and the remaining ten patients had severe anaemia (<8.0 g/dL) (Table 1).

Molecular methods (RT-PCR) found no statistically significant differences with respect to the sex of the patients between the infected vs. non-infected groups (*p* = 0.7517). Likewise, no statistically significant differences were found with respect to the age of the patients between the groups of infected vs. non-infected individuals (*p* = 0.2623). There were no statistically significant differences as to body temperature between the groups of infected vs. non-infected individuals (*p* = 0.1512). The mean haemoglobin concentration in the group of *Plasmodium*-infected patients was found to be 8.1 (2.7) and in the group of non-infected individuals—13.2 (2.7). Thus, the haemoglobin concentration was significantly lower in *Plasmodium*-infected group, compared to non-infected individuals (*p* = 0.0001) (Table 2).

The univariate and multivariate regressions showed that the probability of RT-PCR (+) was higher in patients with a higher body temperature and a lower haemoglobin concentration (Table 3).

In the second study group (i.e., the group consisting of 273 patients admitted to the Amal Hospital on Pemba Island) a total of four *Plasmodium* infections were detected by mRDTs (two *P. falciparum*, two *P. falciparum* + *P. ovale*/*P. malariae*). The positive mRDTs accounted for 1.5% of the tests performed in this group; all positive mRDT results were verified by microscopic examination performed by qualified personnel working at the Amal Hospital. During phase II of the study, the samples were tested using molecular methods (RT-PCR). This testing method demonstrated a total of 13 *Plasmodium* infections (including those infections which had been previously confirmed using mRDTs), and positive RT-PCR test results accounted for 4.8% of all the tests performed. In one case, mRDT detected both *P. falciparum* and Pan species, while RT-PCR in the same sample detected *P. falciparum* only. This discrepancy may be due to the cross-reactivity of the mRDT assay.

The group of *Plasmodium*-infected patients included 12 adults and 1 child. Of all the *Plasmodium*-infected patients, only one person had a normal haemoglobin concentration, three patients had mild anaemia, and nine patients had moderate anaemia (8.0–10.9 g/dL). It is worth pointing out that 10 out of 13 *Plasmodium*-infected patients had never travelled to mainland Tanzania and other malaria endemic areas, which supports the finding that malaria transmission occurs on the Pemba Island in the Zanzibar Archipelago (Table 4).

In the group of patients tested by molecular methods (RT-PCR), there were no statistically significant differences with respect to the sex of the patients between the infected vs. non-infected groups (*p* = 0.6794). Likewise, no statistically significant differences were found with respect to the age of patients between the groups of infected vs. non-infected individuals (*p* = 0.4649). There were no statistically significant differences as to body temperature between the groups of infected vs. non-infected individuals (*p* = 0.8898). Also, no statistically significant differences were found between the two groups with respect to travel to mainland Tanzania (*p* = 0.3040).

The mean haemoglobin concentration in the group of *Plasmodium*-infected patients was found to be 11.1 (1.2), and in the group of non-infected individuals—12.6 (1.9). The haemoglobin concentration was significantly lower in *Plasmodium*-infected vs. non-infected individuals (*p* = 0.0007) (Table 5).

The univariate and multivariate regression showed that the probability of RT-PCR (+) was higher in patients with lower haemoglobin concentrations (Table 6).

## 4. Discussion

Tanzania is one of the most popular tourist destinations for travellers from around the world. Some travellers visit the famous national parks (e.g., Serengeti, Tarangire, Lake Manyara, Kilimanjaro), while others choose to visit the exotic islands of the Zanzibar Archipelago in the Indian Ocean [12]. It needs to be remembered, however, that Tanzania is one of the countries with the highest prevalence of malaria cases and malaria-related mortality [2]. Despite the numerous preventive measures taken, high malaria morbidity and mortality is a major health threat to local residents and hundreds of thousands of foreign visitors alike. In 2022, a total of 6131 laboratory-confirmed malaria cases were reported from the EU countries (with the highest number of cases seen in France, Germany, Spain and Italy), of which 99.8% were travel-related cases, most commonly imported from Sub-Saharan Africa [13]. CDC reports found that although *Plasmodium falciparum* is a dominant in Africa, non-*falciparum* species are also present in the continent [4]. This is a crucial finding in terms of the diagnosis, treatment, and elimination of malaria, including asymptomatic cases. The use of mRDTs designed to detect *P. falciparum* only [14] has resulted in a reduction in *P. falciparum* malaria but was ineffective in limiting the transmission of non-*falciparum* species, which can be found in Africa. This finding has been supported by the results of other screening studies into the prevalence and distribution of *Plasmodium* species in the northern and eastern parts of Tanzania [15,16]. To achieve the eradication and stop the transmission of malaria in endemic areas, it is necessary to select effective diagnostics. Despite many advantages of mRDT (speed of obtaining the result, ease of performance and low price), they can cause false negative results resulting from many reasons (cross-reactions and low parasitaemia) [17]. Although microscopic examination remains the “gold standard” for the confirmation of malaria, molecular methods are becoming increasingly more popular in diagnosing the disease due to their high sensitivity and specificity. Molecular biology testing methods are particularly useful in areas with low-to-moderate malaria transmission, where asymptomatic cases prevail over symptomatic ones, and where the use of mRDTs and microscopic methods is less effective compared to the PCR technique (due to a low parasitaemia). This finding is supported by the results of the present study, as well as the results obtained by other authors [18]. The northern parts of Tanzania, where the country’s major national parks are located, and the Zanzibar Archipelago have become a low-transmission area for *Plasmodium* species. Both of these locations have been extremely popular with tourists from all over the world, in particular with tourists from Europe and North America, i.e., the continents which are free from malaria transmission. Once the COVID-19 pandemic ended, there has been a resurgence in international travel. In Africa, international tourist arrivals have already reached 96% of their pre-pandemic levels [19]. The results obtained emphasise the occurrence of malaria cases both in the mainland and on the archipelago. It is believed that the occurrence of malaria on the islands is most often the result of the importation of the infectious agent from the continent. The residents of Pemba Island with confirmed malaria did not travel to the continent in 77%, which also indicates local transmission. The results obtained indicate the risk of *Plasmodium* transmission both among the local population and among visiting tourists. A large percentage of asymptomatic cases results in a faster spread of the disease and makes its eradication difficult. It is expected that African countries will witness a further growth in the number of international tourist arrivals. Given the fact that malaria is endemic across the continent, including in the most popular tourist destinations, it is crucial to consistently improve the diagnostic methods used for the detection of tropical diseases, as well as to apply effective prevention, including the use of insecticide-treated mosquito nets (ITN). The low economic status of Tanzanians makes it difficult to conduct prophylaxis, and also limits their access to comprehensive diagnostics [20]. The detection and elimination of all malaria cases, including its asymptomatic forms, is important due to the high risk of complications in people with weakened or not fully developed immune systems, especially among pregnant women and children. Although a malaria infection in childhood can lead to the acquisition of immunity, which in adulthood can result in a milder form of disease, the negative effects of infection, especially among young children and patients with chronic diseases, may pose a significant threat to their health and life [21].

## 5. Conclusions

The obtained results demonstrated that the occurrence of malaria in the northern mainland and Zanzibar Archipelago is higher than the official MoH reports present. The study findings are consistent with the reports by CDC, which suggest that non-falciparum species are also present in Sub-Saharan Africa. Molecular biology testing methods are more effective in diagnosing malaria cases in settings with low-to-moderate disease transmission, where asymptomatic cases prevail over symptomatic ones, and where the use of mRDTs and microscopic methods is much less effective, compared to the PCR technique (due to a low parasitaemia). Further studies on the occurrence of malaria and *Plasmodium* species in the population of Sub-Saharan African countries should be conducted to support the diagnosis, treatment and elimination of this disease on the African continent.

## 6. Limitations of the Study

This study had several limitations that could have influenced the main findings. First, the research was conducted in two hospitals only, located in two regions with the lowest malaria incidence, which did not represent the prevalence of the disease in Tanzania. Second, the sociocultural and behavioural factors of the population that could have influenced the occurrence of the disease were not taken into account. Third, *Plasmodium* developmental forms were not included in the microscopic examination, focusing only on the quantitative monitoring of detected infections.

## Figures and Tables

**Table 1:** Patients with positive test results (mRDT or RT-PCR) seen at the Karatu Lutheran Hospital in mainland Tanzania (*n* = 19).

Sex	Age	Temp. (°C)	Hb [g/dL]	mRDT	RT-PCR
F (pregnant)	20	36.5	4.4	negative (−)	P.f.
M	30	36.6	10.9	negative (−)	P.f.
M	47	36.2	3.8	negative (−)	P.f.
F	34	37.3	10.9	negative (−)	P.f.
F	50	36.9	3.9	negative (−)	P.f.
F	24	37.3	9.7	negative (−)	P.f.
M	52	37.2	7.6	negative (−)	P.f.
M	52	36.2	6.6	Pan	P.f., P.o.
F	61	36.5	5.9	negative (−)	P.f.
F	62	36.3	8.2	negative (−)	P.f.
M	70	36.5	7.2	negative (−)	P.f.
F (pregnant)	27	37.0	9.4	negative (−)	P.f.
M	52	36.7	7.5	P.f.	P.f.
F	48	36.3	7.5	negative (−)	P.f.
F	24	36.3	4.4	negative (−)	P.f.
F	23	36.0	10.3	negative (−)	P.f.
F	22	37.5	12.7	P.f.	P.f.
F	60	36.1	11.2	Pan	P.f., P.m.
F	26	36.5	11.0	P.f., Pan	P.f., P.m.

F—female; M—male; Hb—haemoglobin; mRDT—malaria rapid diagnostic test; RT-PCR—real time polymerase chain reaction; P.f.—*Plasmodium falciparum*; Pan—*Plasmodium ovale*/*Plasmodium malariae*; P.o.—*Plasmodium ovale*; P.m.—*P. malariae*.

**Table 2:** Comparison of patient variables admitted to the Karatu Lutheran Hospital, mainland Tanzania (*n* = 449).

Variables	Non-Infected (−) PCR (*n* = 430)	Infected (+) PCR (*n* = 19)	Total (*n* = 449)	*p*-Value
Sex				0.7517 ^1^
female	279 (64.9%)	13 (68.4%)	292 (65.0%)	
male	151 (35.1%)	6 (31.6%)	157 (35.0%)	
Age (years)				0.2623 ^2^
mean (SD)	37.1 (21.9)	41.3 (16.4)	37.3 (21.7)	
range	1.0–96.0	20.0–70.0	1.0–96.0	
median (IRQ)	32.0 (29.0)	47.0 (28.0)	32.0 (29.0)	
95%CI	[35.0; 39.2]	[33.3; 49.2]	[35.3; 39.3]	
Body temperature (°C)				0.1512 ^2^
mean (SD)	36.4 (0.2)	36.6 (0.4)	36.4 (0.3)	
range	36.0–37.4	36.0–37.5	36.0–37.5	
median (IRQ)	36.5 (0.4)	36.5 (0.7)	36.5 (0.4)	
95%CI	[36.4; 36.5]	[36.4; 36.8]	[36.4; 36.5]	
Hb g/dL				<0.0001 ^2^
mean (SD)	13.2 (2.7)	8.1 (2.7)	13.0 (2.9)	
range	3.0–20.2	3.8–12.7	3.0–20.2	
median (IRQ)	13.3 (3.4)	8.2 (5.0)	13.1 (3.6)	
95%CI	[12.9; 13.4]	[6.8; 9.4]	[12.7; 13.2]	

^1^ Chi-square; ^2^ U Mann–Whitney.

**Table 3:** Univariate and multivariate regression of parameters associated with *Plasmodium* infection confirmed by RT-PCR, Karatu Lutheran Hospital, mainland Tanzania (*n* = 449).

	Univariate		Multivariate	
Parameter	OR (95% CI)	*p*-Value	OR (95% CI)	*p*-Value
Sex				
female	1.17 (0.44–3.15)	0.7519		
male	0.85 (0.32–2.29)	0.7519		
Age	1.01 (0.99–1.03)	0.4146		
Body temperature (°C)	9.71 (2.21–42.69)	0.0026	14.16 (2.61–76.82)	0.0021
Hb g/dL	0.60 (0.51–0.71)	<0.0001	0.58 (0.49–0.70)	<0.0001

**Table 4:** Patients with positive test results (mRDT or RT-PCR) seen at the Amal Hospital on Pemba Island (*n* = 13).

Sex	Age	Temp. (°C)	Hb [g/dL]	Travel to Mainland Tanzania	mRDT	RT-PCR
M	28	37.5	11.8	No	P.f.	P.f.
M	55	36.5	12.5	No	P.f.	P.f.
M	22	36.6	13.4	Yes	P.f., Pan	P.f., P.o.
M	33	37.5	12.5	Yes	P.f., Pan	P.f.
F	28	36.7	10.7	No	negative (−)	P.f.
F	34	36.4	10.7	No	negative (−)	P.f.
F	34	36.3	10.8	No	negative (−)	P.f.
F	21	36.6	10.9	No	negative (−)	P.f.
F	21	36.6	10.0	No	negative (−)	P.f.
M	1	36.3	9.4	Yes	negative (−)	P.f.
F	32	36.0	10.5	No	negative (−)	P.f.
F	34	36.2	10.9	No	negative (−)	P.f.
M	67	36.5	10.2	No	negative (−)	P.f.

F—female; M—male; Hb—haemoglobin; mRDT—malaria rapid diagnostic test; RT-PCR—real time polymerase chain reaction; P.f.—*Plasmodium falciparum*; Pan—*Plasmodium ovale*/*Plasmodium malariae*; P.o.*—Plasmodium ovale*.

**Table 5:** Comparison of variables of patients admitted to the Amal Hospital on Pemba Island (*n* = 273).

Variables	Non-Infected (−) PCR (*n* = 260)	Infected (+) PCR (*n* = 13)	Total (*n* = 273)	*p*-Value
Sex				0.6794 ^1^
female	155 (59.6%)	7 (53.8%)	162 (59.3%)	
male	105 (40.4%)	6 (46.2%)	111 (40.7%)	
Age (years)				0.4649 ^2^
mean (SD)	31.1 (19.2)	34.2 (17.0)	31.2 (19.1)	
range	1.0–85.0	1.0–67.0	1.0–85.0	
median (IRQ)	29.0 (24.5)	33.0 (6.0)	29.0 (24.0)	
95%CI	[28.7; 33.4]	[23.9; 44.4]	[29.0; 33.5]	
Body temperature (°C)				0.8898 ^2^
mean (SD)	36.5 (0.3)	36.6 (0.5)	36.5 (0.3)	
range	36.0–37.5	36.0–37.5	36.0–37.5	
median (IRQ)	36.5 (0.3)	36.5 (0.3)	36.5 (0.3)	
95%CI	[36.5; 36.5]	[36.3; 36.8]	[36.5; 36.5]	
Hb g/dL				0.0007 ^2^
mean (SD)	12.6 (1.9)	11.1 (1.2)	12.5 (1.9)	
range	5.1–18.6	9.4–13.4	5.1–18.6	
median (IRQ)	12.7 (2.3)	10.7 (1.4)	12.6 (2.3)	
95%CI	[12.4; 12.9]	[10.4; 11.8]	[12.3; 12.8]	
Travel to mainland Tanzania				0.3040 ^1^
	34 (13.1%)	3 (23.1%)	37 (13.6%)	

^1^ Chi-square; ^2^ U Mann–Whitney.

**Table 6:** Univariate and multivariate regression of parameters associated with *Plasmodium* infection confirmed by RT-PCR, Amal Hospital, Pemba Island (*n* = 273).

	Univariate		Multivariate	
Parameter	OR (95% CI)	*p*-Value	OR (95% CI)	*p*-Value
Sex				
female	0.79 (0.26–2.42)	0.6800		
male	1.27 (0.41–3.87)	0.6800		
Age	1.01 (0.98–1.04)	0.5703		
Body temperature (°C)	2.02 (0.40–10.22)	0.3952		
Hb g/dL	0.68 (0.52–0.89)	0.0048	0.53 (0.38–0.75)	0.0004
Travel to mainland Tanzania				
yes	1.99 (0.52–7.61)	0.3126		
no	0.50 (0.13–1.91)	0.3126		

## Data Availability

The data presented in this study are available upon request from the corresponding author.
